# Do Perineuronal Nets Stabilize the Engram of a Synaptic Circuit?

**DOI:** 10.3390/cells13191627

**Published:** 2024-09-29

**Authors:** Varda Lev-Ram, Sakina Palida Lemieux, Thomas J. Deerinck, Eric A. Bushong, Alex J. Perez, Denise R. Pritchard, Brandon H. Toyama, Sung Kyu R. Park, Daniel B. McClatchy, Jeffrey N. Savas, Michael Whitney, Stephen R. Adams, Mark H. Ellisman, John Yates, Roger Y. Tsien

**Affiliations:** 1 Department of Pharmacology, University of California San Diego, CA 92093, USA; sakinapalida@gmail.com (S.P.L.); pritchard.denise@yahoo.com (D.R.P.); mwhitney5171@gmail.com (M.W.); sadams@health.ucsd.edu (S.R.A.);; 2 National Center for Microscopy and Imaging Research, University of California San Diego, La Jolla, CA 92093, USA; tomdeerinck@gmail.com (T.J.D.); alexjperez@gmail.com (A.J.P.); mellisman@ucsd.edu (M.H.E.); 3 Molecular and Cell Biology Laboratory, Salk Institute for Biological Studies, La Jolla, CA 92037, USA; 4 Department of Molecular Medicine, The Scripps Research Institute, La Jolla, CA 92037, USA; rpark@scripps.edu (S.K.R.P.); dmcclat@scripps.edu (D.B.M.); jeffrey.savas@northwestern.edu (J.N.S.); jyates@scripps.edu (J.Y.III); 5 Department of Neurology, Feinberg School of Medicine, Northwestern University, Chicago, IL 60208, USA; 6 Neurosciences, University of California San Diego, La Jolla, CA 92093, USA; 7 Department of Chemical Physiology, The Scripps Research Institute, La Jolla, CA 92037, USA; 8 Department of Chemistry & Biochemistry, University of California San Diego, La Jolla, CA 92093, USA; 9 Howard Hughes Medical Institute, University of California San Diego, La Jolla, CA 92093, USA

**Keywords:** perineuronal nets, life-long-memory, fear conditioning, SILAM, SBEM

## Abstract

Perineuronal nets (PNNs), a specialized form of extra cellular matrix (ECM), surround numerous neurons in the CNS and allow synaptic connectivity through holes in its structure. We hypothesize that PNNs serve as gatekeepers that guard and protect synaptic territory and thus may stabilize an engram circuit. We present high-resolution and 3D EM images of PNN-engulfed neurons in mice brains, showing that synapses occupy the PNN holes and that invasion of other cellular components is rare. PNN constituents in mice brains are long-lived and can be eroded faster in an enriched environment, while synaptic proteins have a high turnover rate. Preventing PNN erosion by using pharmacological inhibition of PNN-modifying proteases or matrix metalloproteases 9 (MMP9) knockout mice allowed normal fear memory acquisition but diminished long-term memory stabilization, supporting the above hypothesis.

## 1. Introduction

The molecular and cellular basis for very long-term memory is among the most central, captivating, and controversial questions in neuroscience. It requires intra- and extracellular changes that contribute to memory persistence in the brain over time [1,2,3]. Richard Semon coined the term “*engram*” in his theory; these memories leave a physical mark comparable to engraved writing in the brain [4]. We hypothesize that the PNN, a specialized extracellular matrix (ECM), is the substrate that undergoes this physical engraving to stabilize the *engram*. The PNN was first described by Camillo Golgi and Ramon y Cajal (1898), who speculated that the structure might be involved in insulating neurons [5]. Later studies described PNN composition and the development of new antibodies for chondroitin sulfate proteoglycans (CSPGs) [6,7], which were used to investigate individual CSPGs, particularly in the context of glial scar formation [8]. The use of ChABC to disrupt PNN structure by degrading GAGs and thus removing the PNN resulted in memory loss and reopening of critical periods. This suggests that PNNs may regulate experience-dependent synaptic plasticity, maintain remote memories [1,9,10,11,12], and play a role in stabilizing drug addiction [13]. Abnormal PNNs are also correlated with loss or altered memory. PNNs are disrupted in brain regions affected by Alzheimer’s disease, and intact PNNs protect neurons when exposed to toxic β-amyloid deposits [14,15]. Brain tissue samples from schizophrenia and seizure patients show a reduction of PNNs in affected regions [16,17]. Rett syndrome is a severe neurodevelopmental disorder. In brain samples and a mouse model of Rett syndrome, an accelerated maturation of PV neurons and the hyper-formation of PNN structures were described. These findings might be the underlying cause of the Rett syndrome phenotype [18]. These studies demonstrate that a structurally intact PNN is vital to normal brain functions and hints at the possibility that the pattern of holes in the PNN conserves the code for very long-term memory [19].

**Figure 1:**
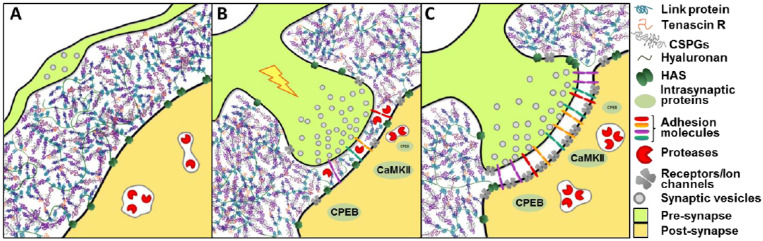
Schematic model of PNN. (**A**) PNN creates a physical barrier that limits synaptogenesis in adult animals. (**B**) Learning-associated synaptic activity leads existing synapses to release matrix metalloproteases from the post-synaptic cell, facilitating synaptic territory enlargement and stabilization. (**C**) A stable and strong synapse with a larger hole in the PNN might support a stronger and long-lasting connection.

The schematic model in Figure 1 illustrates elements of PNNs that may be altered (“edited”) following synaptic activation during memory consolidation or long-lasting memory stabilization. Simply stated, learning-linked synaptic changes involve the release of metalloproteases (MMPs) near the activated synapses, breaking up and eroding adjacent PNN [20].

We suggest that this process might be very local and could result in more permanent “real estate” for the activated synapse, thereby stabilizing and protecting the territory of the larger and stronger synapse [19]. We speculate that the code for memory might reside in the pattern of holes etched in the stable PNN coating, analogous to old computer punch cards where holes permit contact between brushes placed on both sides of the card that allows an electric current flow, like a synaptic connection. Unlike computer punch cards, the hole size in the PNN indicates the allowance of synaptic connection and, hence, the synapse’s strength. This model would require that the stability and longevity of the PNN components be much greater than that of synaptic proteins. To this end, we used SILAM to determine the degradation dynamics of PNN proteins and compared this rate to some known long-lasting proteins histone H4 and myelin basic protein [21]. We also labeled the PNN for imaging with antibodies for aggrecan or with Wisteria Floribunda Agglutinin (WFA). WFA has a high affinity to N-acetyl galactosamine (GalNAc) branches attached to the glycosaminoglycans (GAGs)^12^. WFA staining revealed a thick, fenestrated structure around some neurons in fixed adult brain tissue. This stain specifically labels material surrounding fast-spiking parvalbumin-rich inhibitory interneurons [22,23], as well as a subset of excitatory neurons, such as pyramidal cells in the hippocampal CA2 area, that are associated with social behavior [24,25], in the basolateral amygdala [26] and in the Deep Cerebellar Nuclei. We used structural, proteomic, and behavioral approaches to examine this hypothesis in mice.

## 2. Results

### 2.1. Observation by High-Resolution EM and 3D-EM Confirms That the PNN Holes Are Occupied by Synapses

To test the hypothesis that the pattern of PNN holes holds the code for very long-term memories, we first tested whether synapses occupy all or most of the PNN holes. The above observation has been reported using light microscopy (LM) in cultured neurons [27] and in vivo [26], but this has never been confirmed by high-resolution 3D-EM. 

**Figure 2:**
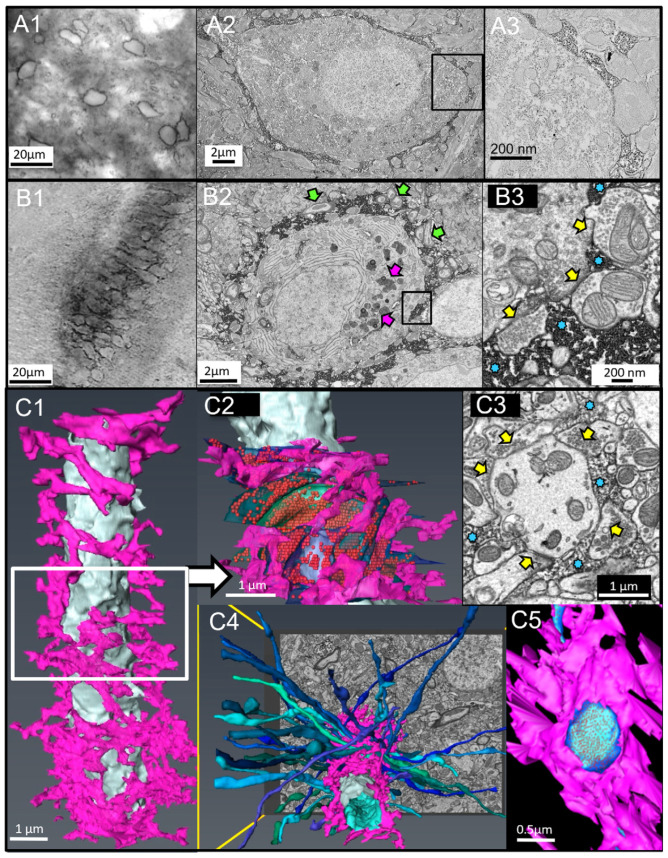
Holes in PNN allocate spaces for synaptic contact. (**A**) The PNNs in the deep cerebellar nucleus were labeled with Ab for aggrecan, followed by biotinylated Ab and streptavidin-HRP. (**A1**) LM of PNN engulfed neurons. (**A2**) EM of a PNN surrounded neuron. (**A3**) Higher magnification demonstrating synapses delimited by PNN. (**B**) Hippocampus CA2 area neurons labeled with WFA-biotin followed by streptavidin-HRP. (**B1**–**B3**) are like (**A1**–**A3**). with the symbols that demonstrate the different components: Green arrows are myelinated fibers, pink arrows are lipofuscin, yellow arrows are synapses, and blue asterisks are PNN. (**C**) Three-dimensional reconstruction of a PNN engulfed dendrite segmented from a serial block face scanning electron microscope volume. (**C1**) Dendrite is colored white, and PNN is in fuchsia. (**C2**) Synaptic boutons are highlighted in green and blue, with red synaptic vesicles showing through the transparent plasma membrane. The segmentation highlights only the midsection of the dendrite volume. (**C3**) An EM image from the center of this area demonstrates the dendritic membrane, surrounded by either synapses or PNN. The PNN was identified by its higher osmophilic property and appeared as granular matter surrounding PNN-engulfed neurons. (**C4**) Top view of the dendrite with axons synapsing onto the mid-section. (**C5**) A view from the inside of the dendrite through a PNN hole demonstrating a single synapse contacting the dendrite.

We applied advanced 3D-EM imaging technologies to examine the relationship between synapses and PNN holes [23]. Using antibodies (Ab) for aggrecan in the deep cerebellar nucleus (Figure 2(A1–A3)) and WFA-biotin in the hippocampal CA2 region (Figure 2(B1–B3)), we heavily labeled PNN-engulfed neurons that exhibited openings that are only occupied by synapses (Figure 2(A3,B3)). To obtain a better understanding of the PNN-synapse relationship, we used serial block-face scanning electron microscopy (SBEM) to create high-resolution 3D-EM [28]. We manually segmented the plasma membrane and semi-automatically marked the PNN of neurons and dendrites from a hippocampal CA2 volume. In Figure 2(C1), the dendrite (white) is surrounded by the porous PNN (magenta).

We traced all the presynaptic boutons (translucent greens and blues) and neurotransmitter vesicles (red balls) within the center section of the dendrite. Interestingly, more than 98% of the dendrite plasma membrane was in contact with PNN or presynaptic boutons. We did not observe contacts with other passing dendrites, myelinated fibers, or astrocytic processes (Figure 2(C2)). This contrasts “naked” dendrites without a PNN coating, which have membrane-to-membrane contact with myelinated fibers or other passing dendrites (Figure S1). 

The single-plane EM image in Figure 2(C3) demonstrates that the entire circumference of the dendrite is occupied by either PNN or synapses that, in turn, are restricted and protected by PNN. A three-dimensional view of the axons that synapse onto the PNN-engulfed dendrite is presented as a volume in Figure 2(C4). Figure 2(C5) is a view from inside this dendrite through a hole in the PNN, exhibiting synaptic contact that is confined and secured by the surrounding PNN. This supports the idea that to increase and thus strengthen a PNN-surrounded synapse, the PNN needs enzymatic chiseling.

### 2.2. PNN Protein Longevity

As some memories can last for an organism’s lifetime, we proposed that the substrate that holds the code for memory should be stable and have a slow turnover. Other hypotheses for the preservation of remote memory postulate that key synaptic proteins, including calcium–calmodulin (CaM)-dependent protein kinase II (CaMKII) [2], protein kinase M-zeta (PKMζ) [29], or cytoplasmic polyadenylation element binding protein (CPEB3) [30] store memory by passing the information to all subsequent generational copies of the protein aggregates within synaptic processes. For all the proteins that are suggested as memory mediators by these hypotheses, faithful repetitive intergenerational copying would be necessary to maintain a steady state over time. 

**Figure 3:**
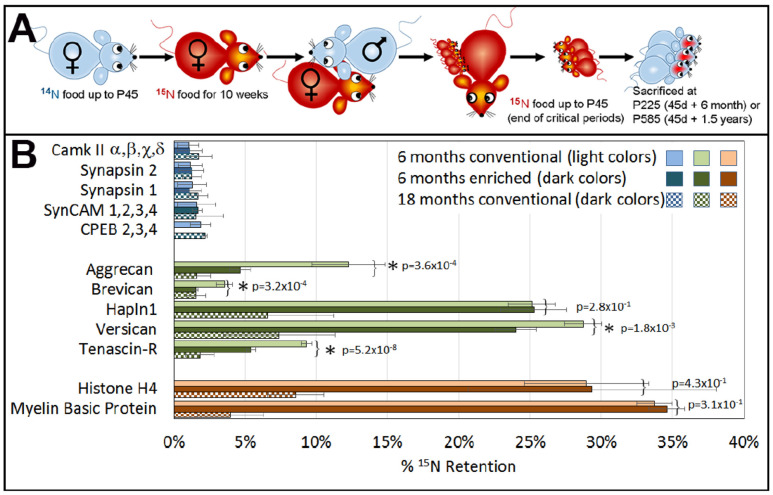
The pulse-chase SILAM experiment demonstrated that PNN proteins are long-lived, and an enriched environment accelerates their erosion. (**A**) Schematic representation of the experiment that is detailed in the Supplementary Materials. (**B**) MuDPIT results demonstrate retention of 15N in stable proteins compared to proteins with fast turnover rates. Light and dark color bars represent 6-month pulse-chase in conventional and enriched environments, respectively; 18-month pulse-chase bars are checkered. Histone-H4, and Myelin Basic Protein (MBP) are known stable proteins and serve as a reference (orange). Synaptic proteins (blue) show low ^15^N fractional abundance even at the 6-month chase, while PNN proteins (green) retain a high level of ^15^N fractional abundance that is comparable to histone-H4 and MBP at 6 months pulse-chase. Some PNN proteins retained significant ^15^N fractional abundance even at the 18-month chase. A comparison of protein longevity between EE and CC shows that synaptic proteins (checkered dark blues) did not demonstrate differences in turnover. PNN proteoglycans were eroded more in EE mice compared to littermates in CC, except for Hapln1 (checkered dark greens). In contrast, there was no change in known MBP and histone-H4 (checkered dark oranges). The number of peptides included in each group is detailed in Table S1.

To test the differential stability of synaptic and PNN components in the brain, we designed a SILAM experiment [31], fully labeling mice proteins with ^15^nitrogen (^15^N) for proteomic analysis. A schematic representation of the experimental design is detailed in Figure 3A and further described in the Supplementary Materials. 

The ^15^N pulse was chased by ^14^N starting at P45 (pulse-chase method). Mice were sacrificed at P45, P225, and P585 (0, 6, and 18 months-^14^N chase). We prepared synaptosomes and PNN-enriched fractions at each harvest point and performed multi-dimensional protein identification technology (MudPIT) analysis to identify brain proteins’ ^15^N retention. Previous SILAM studies primarily focused on identifying intracellular proteins, while several extracellular proteins were also identified to be highly stable. The previous analysis was conducted in cultured neurons and included Tenascin-C, Tenascin-R, agrin, and lamin [32,33]. In brain tissue, the following long-lasting molecules were identified: versican, Hapln1, and MBP [33,34]. 

We analyzed synaptic, PNN, and known long-lasting proteins. At the 6-month pulse-chase point, the synaptosomal proteins CaMKII, synapsin, adhesion molecules (SynCAM), and CPEB did not meet the criteria of ^15^N fractional abundance for long-lived proteins, as described in the material and methods section. 

We averaged all isoforms of CaMKII, SynCAM, and CPEB to achieve statistically meaningful data points (Figure 3B, light blue bars 6 months of pulse-chase). Meanwhile, constituents of the PNN, aggrecan, brevican, tenascin-R, versican, and Hapln1 retained 5–28% ^15^N fractional abundance (Figure 3B light-green bars). We compare these data to proteins that are known to have a slow turnover, like histone-H4 and myelin basic protein (MBP), that retained 28–34%, respectively (Figure 3B, light-orange bars). This is comparable to the 7–37% ^15^N that was previously found in other long-lasting structural proteins such as collagens and lamins [34]. 

In aged mice at 18 months pulse-chase, the PNN components were still clearly detectable at 2–7% ^15^N retention (Figure 3B, dark-green checkered bars). This was comparable to MBP and histone-H4, whose ^15^N had declined to 4% and 8%, respectively (Figure 3B dark-orange bars). PNN components, MBP, and histone-H4 demonstrate a very slow turnover at the 18-month time point, while synaptic proteins maintained the same levels of ^15^N retention as in the 6-month pulse-chase points (Figure 3B dark-blue checkered bars). This further shows that synaptic protein values at both 6 and 18 months pulse-chase could not be considered as “heavy”. Hence, some of the PNN components are comparable to known long-lasting proteins in the brain whereas synaptic proteins have rapid turnover. 

The 6-month pulse-chase group was divided into an enriched environment (EE, see below) and conventional cages (CC). We speculated that the mice in the EE—with changing toys, spinning disks, mirrors, balancing beams, tunnels, occasional music, scents, and frequent handling—would activate the brain and induce learning and memories that the CC group could not acquire. Thus, PNN erosion would result in less ^15^N retention in PNN proteins in EE-caged mice. Indeed, we observed a statistically significant reduction in ^15^N retention in brains from EE mice (Figure 3B light and dark green bars) compared to the CC group (2-tailed *t*-test). 

The known long-lived proteins that are represented here as a reference (histone-H4 and MBP) had the same turnover rate in EE and CC groups. This further validates the authenticity of the differences in PNN longevity between EE and CC. However, Hapln1 turnover was not accelerated due to EE, which might point to the preservation of the scaffolding of the hyaluronic acid net with Hapln1 tightly anchored. 

### 2.3. Remote-Memory Stabilization Requires MMP(s) Activity

PNN can be eroded by specific proteases that either create new holes for synaptogenesis or enlarge existing holes to expand the synaptic contact territory and consequently strengthen synaptic input [35]. The main proteases involved in this activity are MMP 2 and 9. 

Fear conditioning has been demonstrated to evoke the expression of MMP-9 mRNA and enzyme activity in three major mouse brain structures: the hippocampus, prefrontal cortex, and amygdala [36]. To test the role of MMPs in establishing long-lasting memories, we modified protease activity via pharmacological inhibition or genetic knockout (KO). We performed classic fear conditioning, assessing the association of cues (tone and light) with an adverse mild electric shock. 

Tests were performed 24 h and 4 weeks after conditioning. We compared memory recall in four groups of wild-type (WT) and MMP9 KO mice, and each group was injected with either a broad-spectrum MMP inhibitor, prinomastat (AG-3340), or with the vehicle (DMSO). Prinomastat has been demonstrated to cross the blood-brain barrier (BBB) [37] and to inhibit MMP-2, -3, -9, -13, and -14 [38]. We also tested the presence of prinomastat in the brains of injected animals to verify that the drug was indeed crossing the BBB. Tests were performed at 0, 2, 4, 8, and 24 h after IP injection of 4 mg prinomastat in 20 µL DMSO. We used the MMP2 FRET assay [39] (Figure S2). We found more than 250 nM of prinomastat in brains 2 h post-injection. The concentration of prinomastat decreased with time to a level of close to 100 nM 24 h after injection.

**Figure 4:**
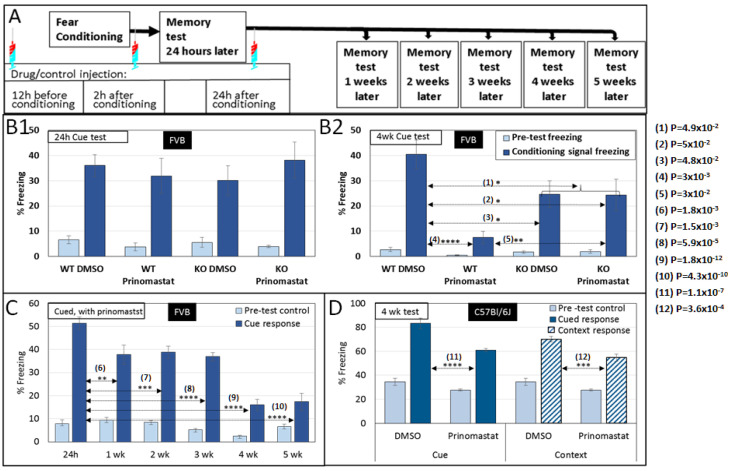
MMP inhibition affects remote memory but not memory acquisition. (**A**) Schematic representation of the behavior experiment. Mice were injected IP with 4 mg prinomastat dissolved in DMSO: 12 h before fear conditioning, 2 h after fear induction, and 2 h after the memory recall test (24 h test). In most experiments, a memory test was performed 4 weeks later. Each group consisted of 12 mice of mixed gender, and each experiment was repeated 3 times. (**B1**) IP injection of the broad-spectrum MMP inhibitor; prinomastat did not affect fear-conditioning acquisition; tested 24 h after induction in either FVB WP or FVB MMP9 KO. (**B2**) A memory test 4 weeks later revealed a highly significant difference between the control group injected with the vehicle (DMSO) and the experimental groups. MMP9 KO also demonstrated a significantly lower freezing percentage in the 4 weeks’ test compared to the wt. MMP9 KO did not exhibit a difference between the DMSO and prinomastat-injected groups in the remote-memory test. (**C**) Testing the dynamics of prinomastat on memory indicates an increasing impairment with time. (**D**) Prinomastat-impaired remote-memory in C57BL/6J mice in both cued and contextual tests.

A cue test performed 24 h post-induction indicated that fear conditioning was equally acquired in all the groups. Nevertheless, testing the mice four weeks later revealed a large statistically significant difference in memory retention between the WT that were injected with DMSO vs. prinomastat (2-tailed *t*-test *p* < 10^−4^; Figure 4(B2)). We concluded that MMP inhibition might have prevented the long-lasting memory, more likely at the consolidation or stabilization stage rather than at the recall. 

The MMP9-KO mice had a significant deficiency in memory at the four-week test, compared with the WT or 24 h post-induction test. However, the KO mice memory at the four-week test did not differ between the DMSO and the prinomastat-injected group. It is possible that the partial memory in MMP9-KO mice in the 4-week test might be due to life-long compensation with prinomastat insensitive proteases because of the lack of MMP9 in their system (Figure 4(B2)). 

We also tested the dynamics of remote memory in prinomastat-injected mice. Separate groups of mice were tested 1, 2, 3, 4, and 5 weeks after fear induction. We found decreased memory, which seemed to plateau at 4 weeks after induction (Figure 4C). This might be due to fading recent memory, while the consolidation/stabilization of remote memory was impaired by MMP inhibition. Control groups injected with DMSO exhibited normal remote memory (Figure S3).

Finally, we tested MMPs’ inhibition effect on memory consolidation/stabilization in C57Bl/6J, which are known to have a better learning ability compared to FVB mice [40]. Our findings demonstrate that C57Bl/6J results were similar to FVB mice. Prinomastat did not affect the acquisition of fear induction, as demonstrated in the 24 h test (Figure S4A). However, there was a significant effect on both cue and context tests 4 weeks after conditioning (Figure 4D). FVB demonstrated minimal contextual memory even 24 h after fear induction (Supplementary Figure S4), while C57Bl/6J demonstrated greater memory in both cue and contextual tests (Figure 4D). Overall, we observed that PNN proteolysis inhibition did not affect acquisition/recent-memory recall (24 hr) but greatly lowered remote memory when tested 4 weeks after acquisition.

The temporary inhibition of MMPs with prinomastat followed the schedule and dosage described in Figure 4A, maintaining sufficient MMPs inhibition to prevent memory consolidation/stabilization. An alternative injection schedule of omitting one or two of any of the injections or lower prinomastat dosage (2 mg) was less or not effective. Moreover, a higher dosage (8 mg or 16 mg) did not change the degree of memory deficiency. Therefore, our data might be compatible with memory stabilization onto PNN-modulation-dependent storage.

The 2 h post-retrieval prinomastat injection was required for remote memory impairment, which suggested that MMPs are essential for post-retrieval consolidation into the brain area of the lasting memory location. However, when a single prinomastat dose was injected 2 h post-retrieval, there was no remote memory impairment.

A weekly memory test after prinomastat injection demonstrated a gradual loss of remote memory (Figure 4C). These results support the hypothesis that PNN erosion is necessary for remote memory stabilization. 

## 3. Discussion

The conservation of life-long memories is crucial for sustaining vital physiological functions and retaining important information acquired as animals interact with their environment. Once established, conservation relies on the stability of synaptic input within neural circuits over extended periods. Considering that most intracellular proteins undergo rapid turnover, including those proposed as memory substrates [32,33]. It is reasonable to speculate that an extracellular structure with greater stability, such as the perineuronal net (PNN), may play a pivotal role in defining, stabilizing, and safeguarding synaptic connections and their territory. Therefore, PNNs could potentially serve as a structural scaffold that preserves the memory *engram*.

To investigate the PNN’s role as a stabilizer, we analyzed the relationship between PNN structure and synapses using electron microscopy (EM) and 3D serial block-face scanning electron microscopy (SBEM). Our findings demonstrate that PNN perforations are occupied by synapses.

We employed the SILAM method to assess PNN protein turnover dynamics. Our results indicate prolonged stability of PNN proteins, contrasting with the short lifespan of synaptic proteins.

Furthermore, mice subjected to an enriched environment that started at the end of the ^15^N labeling pulse exhibited accelerated PNN degradation compared to littermates housed in a conventional non-stimulating environment. We propose that environmental enrichment promotes learning-induced synaptic expansion, necessitating the enlargement of PNN perforations to accommodate and safeguard these synapses [36].

Moreover, we investigated fear-conditioning acquisition and retention. Our findings revealed that preventing PNN erosion either through the use of a pharmacological agent to inhibit MMP activity or by employing MMP9 knock-out mice did not impair fear-conditioning acquisition. However, there was a notable deficiency in fear-conditioning remote memory. These results bolster our hypothesis regarding the PNN’s role in stabilizing the code underlying remote memory. Furthermore, our study lays the groundwork for future exploration of the PNN’s involvement in age-related and neurological memory impairment processes, such as Alzheimer’s disease [14,15], Rett syndrome [18], schizophrenia, and seizure patients [16,17].

Therefore, we propose that the PNN plays a crucial role in preserving memories in our brains for a lifetime. This is achieved through its function in protecting, defining territories, and stabilizing synapses. Our argument is supported by a range of observations and experiments, including the significant memory erasure that follows the enzymatic degradation of the PNN using chondroitinase [9,10,11,41]. Another key aspect of our argument is the establishment of the PNN structure at the closure of critical periods. This process, we suggest, is instrumental in memory preservation and the maintenance of synaptic stability [42,43] and correlating infantile amnesia to the end of PNN deposition at the closure of critical periods [44,45,46]. 

Pyramidal neurons in the hippocampus lack PNNs and can be easily modulated [26]. However, pyramidal neurons in the hippocampal CA2 region are heavily engulfed in PNN and resist plasticity [47]. 

An important element of a PNN is its stability once it has been deposited and its resistance to proteases other than MMPs. It has also been demonstrated that brevican neoepitopes that result from enzymatic degradation can be found at synapses and coincide with an increase in synaptic size and strength [48]. This indicates that PNN erosion is associated with synaptic strengthening and larger synaptic territory. Our hypothesis suggests that the PNN pattern of holes is a better substrate for stabilizing the *engram*. This is supported by our SILAM studies exploring the PNN component’s longevity [32] compared to the shorter-lived intracellular synaptic protein (median turnover rate of less than 4 days). 

We found that some PNN proteoglycans have the same ^15^N retention as known stable proteins, like histone H4 and MBP. Several synaptic proteins have been implicated in memory maintenance, including PKMζ, CPEB, CaMKII, and Arc/Arg2.1. However, these proteins have only been examined in relatively recent memory models and turnover on the time scale of hours to days, suggesting a regulatory role rather than a long-term structural one [2,29,33,49,50]. 

Intracellular proteins are also susceptible to cellular or brain-wide disruptions that do not necessarily affect memory, including seizure, barbiturate overdose, or metabolic stress, suggesting that they are unreliable substrates for encoding the *engram*. Extracellular proteins remain sequestered away from intracellular ubiquitin and proteasome-mediated degradation and are thought to be less susceptible to disruption. 

The mice that experienced an enriched environment (EE) created a lasting memory of new experiences, including auditory, tactile, motor skills, social, and olfactory stimulation. The creation of these experiences and memories might have eroded PNNs in many parts of the brain, providing a bigger territory for the potentiated and presumably larger/stronger synapses, as well as new synaptic connections [41]. This may explain the lower ^15^N retention of long-lived PNN/ECM components compared to their littermates that lived in conventional cages (Figure 3B, light vs. dark bars). In contrast, in the behavioral experiment, we prevented PNN editing and modulation by blocking MMP activity during learning and consolidation/stabilization.

In contrast, in the behavioral experiment, we prevented PNN editing and modulation by blocking MMP activity during learning and consolidation/stabilization. 

The results of the SILAM labeling confirmed that the PNN/ECM contains extremely stable proteins, as expected from a component that might stabilize the *engram*. The potential removal of ^15^N labeled proteoglycans, subsequently replaced by newly synthesized molecules, could produce enlarged PNN holes. Confirmation of this hypothesis requires microscopic visualization of changes in PNN hole size in an area that undergoes a learning process or a double-pulsed SILAM experiment using two different stable isotopes.

Varieties of proteases conduct activity-dependent PNN erosion. The proteases required for PNN remodeling have been extensively implicated in synaptic plasticity and LTP. Several groups (see review [51]) have demonstrated that MMP inhibition using antisense oligonucleotides, broad-spectrum pharmacological inhibitors, and neutralizing antibodies can prevent late-phase LTP in various brain regions. Intracerebroventricular injection of a broad-spectrum MMP inhibitor has been reported to attenuate water maze learning for many days after injection. Furthermore, it has been reported that PNN plays a role in regulating fear memory plasticity in the amygdala and visual cortex [1]. PNN formation increases in the amygdala between P16 and P45, an interval corresponding to the establishment of fear memories that are resistant to extinction and are, therefore, more persistent [10,24]. In an experiment that is a reverse of our MMPs’ inhibition, Gogolla and co-workers [10] removed the PNN by locally injecting ChABC into the basolateral amygdala (BLA), enabling the extinction of fear memory resembling the extinction effect on young animals at P16 prior to the end of PNN deposition in the amygdala [9].

Here, we report that the broad-spectrum MMP inhibitor prinomastat can impair long-lasting memory stabilization. This action could be mainly mediated through the inhibition of MMP9 because MMP9 knockout mice had a significantly lower ability to recall fear conditioning 4 weeks after induction, yet no further reduction in memory was induced by prinomastat (Figure 4B(B1,B2)). 

The temporary inhibition of MMPs with prinomastat might be compatible with memory stabilization onto PNN-modulation-dependent storage. The requirement for a 2 h post-retrieval prinomastat injection for remote memory impairment might suggest that MMPs are required for post-retrieval consolidation into the brain area of the lasting memory location, which might start at the time that the memory is acquired. Possibly, memories are rapidly encoded into dynamic intrasynaptic molecules that are gradually diminished unless they have been consolidated into stable and permanent storage by forming or enlarging holes in the PNN. These shorter-term memories did not require prinomastat-sensitive MMP activity. Experiments on human subjects conducted by Bach et al. [52,53] also demonstrate that MMP inhibition reduced associative memory. Bach et al. blocked MMP-9 with doxycycline, showing that MMP-9 is required for structural synapse remodeling involved in memory consolidation. They suggested using this treatment as a therapeutic target for unwanted aversive memories since the MMP-9 inhibition attenuated human threat conditioning.

Our results propose some answers to the enigma of *engram* stability and memory conservation. By providing a protected “real estate”, the PNN holes’ location and size might provide the code for life-long memories.

## 4. Materials and Methods

Reagents: The primary antibody used rabbit polyclonal anti-component (Millipore, Darmstadt, Germany), and HRP-conjugated mouse secondary antibody (Cell Signalling, Danvers, MA, USA) was used at 0.4 µg/mL. Fluorescein-conjugated Wisteria floribunda agglutinin (WFA) and WFA-biotin (Vector Labs, Burlingame, CA, USA) were used at 10 µg/mL. Streptavidin–HRP (VECTASTAIN^®^ Elite^®^ ABC-HRP). FVB wild-type, MMP-9 knockout (FVB background), or C57Bl/6J mice were purchased from Jackson Laboratory and bred at the UCSD vivarium.

### 4.1. Electron Microscopy

For this study, 7.5-month-old FVB mice were anesthetized and perfused with normal Ringer’s solution at 35 °C followed by 0.15 M cacodylate buffer containing 4% formaldehyde (Electron Microscopy Sciences, Hartfield, PA, USA). Brain tissue was cut into 100 µm vibratome sections. The tissue slices were placed in cold cryoprotectant (78% 0.01 M PBS, 20% DMSO, 2% glycerol) for 10 min and then they were rapidly plunged into liquid nitrogen for several seconds (until they turned white). Then they were re-immersed in cryoprotectant and allowed to thaw. This cycle was repeated 3 times with the last cycle thawed in cacodylate buffer. We blocked the tissue’s natural avidin and biotin following the Vector Laboratory protocol. The tissue was then labeled with anti-aggrecan antibody (Millipore) or WFA-Biotin (Vector Laboratories, Newark, CA, USA) (20 µg/mL in cacodylate buffer) overnight on a slow shaker at 4 °C followed by 3 × 5 min wash. The primary Ab was followed by a secondary goat anti-rabbit Ab conjugated to HRP (BioRad) or the WFA-biotin was reacted with streptavidin-HRP (Vector Laboratories). This was followed by 2 min of incubation of the slices in 0.15 M cacodylate buffer containing 2.5% glutaraldehyde (Polysciences), rinsed 5 × 2 min in chilled buffer, and then treated for 5 min in buffer containing 20 mM glycine to quench unreacted glutaraldehyde, followed by 5 × 2 min rinses in chilled 0.15 M cacodylate buffer. The HRP reaction consisted of a freshly diluted solution of 0.5 mg/mL (1.4 mM) 3,3′-diaminobenzidine (DAB) tetrahydrochloride, or the DAB free base (Sigma, Tokyo, Japan) dissolved in HCl was combined with 0.03% (*v*/*v*) (10 mM) H_2_O_2_ in chilled 0.15 M cacodylate buffer, and the solution was added to cells for 1 to 15 min, depending on the sample [54]. The DAB solution was removed to end the reaction, and cells were rinsed for 5 × 2 min with chilled 0.15 M cacodylate buffer. For transmitted EM, the slices were incubated in 2% osmium tetroxide (Electron Microscopy Sciences) for 30 min in a chilled buffer and then rinsed 5 × 2 min in chilled distilled water. Slices were incubated overnight at 4 °C in 2% aqueous uranyl acetate (Electron Microscopy Sciences). The slices were then dehydrated in a cold-graded ethanol series (20%, 50%, 70%, 90%, 100%, 100%) for 2 min each and rinsed once in room temperature anhydrous ethanol to avoid condensation. Durcupan ACM resin (Electron Microscopy Sciences) infiltration was conducted using 1:1 (*v*/*v*) anhydrous ethanol and resin for 30 min, then 100% resin 2 × 1 h, then it was put into fresh resin and polymerized in a vacuum oven at 60 °C for 48 h. Areas of interest were identified by transmitted light and were sawed out and mounted on dummy acrylic blocks with cyanoacrylate adhesive (Krazy Glue, Elmer’s Products, Westerville, OH, USA). The block was trimmed, and ultrathin (80 nm thick) sections were cut using an ultramicrotome (Leica Ultracut UTC6). The sections were imaged on a JEOL 1200 TEM operating at 80 keV.

For Serial Block Face Scanning EM, the following SBEM protocol was used: 

### 4.2. SBEM

Mice were anesthetized and perfused with normal Ringer’s solution containing xylocaine (0.2 mg/mL) and heparin (20 U/mL) for 2 min at 35 °C followed by 0.15 M cacodylate buffer containing 2.5% glutaraldehyde (Polysciences) and 2% formaldehyde (Fisher Scientific, Hampton, NH, USA) with 2 mM CaCl_2_ at 35 °C for 5 min. Brains were removed and postfixed for 18 h at 4 °C in the same solution. Brain tissue was cut into 100 µm thick sections using a vibratome (Ted Pella) in ice-cold 0.15 M cacodylate buffer containing 2 mM CaCl_2_ and then washed for 30 min in the same solution. Tissue was placed in a solution containing 1.5% potassium ferrocyanide (Electron Microscopy Sciences) in 0.15 M cacodylate buffer with 2 mM CaCl_2_ 2% and aqueous osmium tetroxide for 1 h at room temperature (RT). The tissue was processed by placing it in 1% filtered thiocarbohydrazide (Electron Microscopy Sciences) for 20 min at R, 2% osmium for 30 min at RT, and 1% uranyl acetate overnight at 4 °C following triple rinses in DDH_2_O at RT for 30 min. The tissue was triple rinsed in DDH_2_O for 5 min between each step. We prepared lead aspartate solution (0.066 g lead nitrate; Electron Microscopy Sciences) by dissolving it in 10 mL of 0.003 M aspartic acid solution, with the pH adjusted to 5.5 with 1 N KOH, and warmed in a 60 °C oven for 30 min and filtered. Sections were placed into filtered lead aspartate solution in the 60 °C oven for 30 min. The tissue was rinsed five times for 3 min in DDH_2_O and then dehydrated through graded alcohols into acetone and flat-embedded in Durcopan resin (Electron Microscopy Sciences) between mylar strips and placed in a 60 °C oven for 48 h. 

Resin-embedded tissue was mounted on aluminum specimen pins (Gatan) using cyanoacrylate glue and precision trimmed with a glass knife to a rectangle ∼0.5 × 0.75 mm, so the tissue was exposed on all four sides. Silver paint (Ted Pella) was used to electrically ground the edges of the tissue block to the aluminum pin. The specimen was then sputter-coated with a thin layer of gold/palladium to enhance conductivity. After the block was faced with a 3View ultramicrotome unit (Gatan) to remove the top layer of gold/palladium, the tissue morphology became visible by back-scattered electron detector imaging, and the remaining coating on the edges of the block served to reduce charging. A low-magnification image (∼500×) was collected to identify neurons engulfed with PNN for serial image collection. Tissue blocks were scanned using a Quanta field emission gun scanning electron microscope (FEI) or a Merlin scanning electron microscope (Zeiss) and sectioned at 70 or 40 nm thickness. 

### 4.3. Image Reconstruction

The 3D reconstruction of the images obtained with the SFBSEM was conducted in two modes: a manual tracing of membranes and organelles in each slice and a semi-automatic segmentation that is described in [55]. We used iMod software, and the final image was reconstructed using Amira software. 

### 4.4. Stable Isotope Labeling in Mammals (SILAM)

Four FVB females were fed ^15^N-based spirulina chow (Cambridge Isotope Laboratories, Inc., Cambridge, UK) starting at P45 for ten weeks. A male was introduced for one week for mating, and the females were kept on a ^15^N chow diet during gestation and lactation. At P21, the pups were weaned and were kept on a ^15^N diet up to P45 and then switched to a ^14^N normal chow. The pulse-chase was terminated at different times (months: 0 = P45, 6 months = P225, 18 months = P545). For each data point, the brain of a male and female was removed quickly into ice-cold PBS. The brains were cut in half, weighed, and half processed for synaptosomes enrichment and the other half for PNN enrichment. For the 6-month period, a group of 4 P45 pups was transferred into a large cage containing toys, a treadmill wheel, mirrors, frequent handling, and extra tactile, auditory, and olfactory stimuli to enhance and enrich their experience. 

#### 4.4.1. Synaptosomes Enrichment

Ten volumes *w*/*v* of 0.32 M sucrose (Sigma-Aldrich, St. Louis, MO, USA), 4 mM HEPES (Sigma-Aldrich) buffer (pH57.4), and cocktail inhibitor tablets (Roche Diagnostics Indianapolis, IN, USA) were added to the brain tissue before homogenization with a Teflon pistol attached to a tissue homogenizer rotating at 900 rpm. The tube was slowly moved (up/down) 10–15 times until the solution appeared homogeneous. The homogenate was centrifuged twice at 1000× *g* for 10 min to pellet the nuclei, and the two supernatants were combined and centrifuged at 10,000× *g* for 15 min. The pellet was suspended and centrifuged again at 10,000× *g* for 15 min. This pallet was used as the synaptosome-enriched fraction. 

#### 4.4.2. PNN Enrichment

Brains were homogenized on ice in a glass homogenizer tube in solubilization buffer (10 mM TrisHCl pH 7.5, 150 mM NaCl, 0.5% Chaps, protease inhibitor cocktail (complete, Mini, EDTA-free Protease Inhibitor Cocktail Tablets, Roche) 1 tablet/50 mL) using a Teflon pestle at 900 RPM with 10–15 slow strokes until the tissue looked homogenized. Then, the tissue homogenate was sonicated (Sonic Dismembrator 500) on ice (30 s at 30%) four times. Then, the homogenate was centrifuged at 3900× *g* for 30 min in a Sorvall SLA-1500 rotor. The supernatant was then ultracentrifuged at 28,000× *g* for 30 min using a Sorvall SLA-1500 rotor. According to Vector lab protocol, the resulting supernatant was loaded on a WFA lectin chromatography (Vector Lab, CA, USA). The eluate containing the enriched PNN was concentrated by centrifugation in an Amicon Ultra 4, 10 K cutoff for 15 min in a swing bucket at 4500 RPM in a Backman countertop centrifuge (Alegra X-22R rotor). This protocol was adapted and modified from Pacharra S. et al. [56].

### 4.5. MudPIT and LTQ Velos Orbitrap MS

Proteomics of PNN and synaptosomes’ enriched fractions from brains of the ^15^N pulse-chase experiment are described in detail in Butko et al. (2013) [57]. 

### 4.6. Long-Lived Protein Identification

This is described in detail in Toyama et al. (2013) [25]. In short, we identified long-lived proteins in brain fractions (PNN or synaptosomes enriched fractions) using average peptide enrichments (APE) and a Python script to filter the data. Peptides with profile scores < 0.8 were eliminated. Calculation of ^15^N fractional abundance had to be greater than 2.5% to classify a peptide as “m”. All peptides with profile scores > 0.8 but <2.5% ^15^N were considered “light”. Proteins were considered long-lived if they had more than two “heavy” peptides. More than 65% of the peptides for that protein were “heavy”, and the average ^15^N for all peptides from that protein was >2.5%. Proteins with a single ^15^N-enriched peptide were discarded as probable misassignments

### 4.7. Fear Conditioning

Littermates FVB wild-type, MMP-9 knockout (FVB background), or C57Bl/6J mice were purchased from Jackson Laboratory and bred at the UCSD vivarium. Each experimental group contained 12 mice of mixed gender, and each experiment was repeated 3 times. Fear conditioning was induced at 10 weeks of age using Med Associate Inc. equipment. Mice were introduced into the chamber for 3 min of acclamation to the new environment (pre-test freezing evaluation was done during this time). This was followed by a white light and tone (80 dB) for 30 s with a scrambled foot shock of 0.8 mA at the last second. The light, tone, and shock were repeated 3 times at 1 min intervals. The mice were removed from the chamber 1 min after the last shock. Mice were injected with 20 µL of either vehicle (DMSO) or 4 mg of prinomastat hydrochloride (Sigma-Aldrich catalog # PZ0198) in 20 µL DMSO 12 h prior to fear conditioning, 2 h after fear conditioning, and 2 h after the 24 h retrieval test. Retrieval testing was conducted 24 h and 4 weeks post-conditioning. For the freezing evaluation, we used video analysis software from Med Associate Inc. with freezing parameters of six frames and a threshold motion of 30. Data analysis was conducted by combining the results from the 3 separate experiments for each condition. Freezing average, standard error, and *p* values (2-tailed *t*-test) were conducted using the Excel program. The data where the animals that were involved in fights (mostly males) after induction or demonstrated health issues (ear infection) were excluded from the retrieval testing and were not included in the statistical analysis. 

### 4.8. MMP2 FRET Assay to Determine Whether Prinomastat is Crossing the BBB

Mice were injected IP with 4 mg prinomastat, and their brains were removed 2, 4, 8, and 24 h after injection and stored at −80 °C until the brains were processed. Brains were homogenized in MMP assay buffer, boiled for 10 min, and debris was spun out. The supernatant was then sequentially filtered through a 0.2 µm, and then 100 K, 30 K, 10 K, and 3 K cutoff columns (Amicon) before using the MMP2 FRET assay as described in detail in Hingorani et al. (2017) [39]. Assays were conducted with a 30× dilution of extracts, and the prinomastat concentration was determined by running a calibration curve using known prinomastat concentrations (Figure S2A).

## Data Availability

https://massive.ucsd.edu/ProteoSAFe/dataset.jsp?task=0888173f1f6541ce878d4875f0154f25.

## Supplementary Materials

The following supporting information can be downloaded at: https://www.mdpi.com/article/10.3390/cells13191627/s1, Figure S1: PNN engulfed dendrite surrounded with PNN and synapses. Myelinated fibers that pass by do not touch the dendrite; Figure S2: MMP2 FRET Assay to Determine Whether Prinomastat is Crossing the BBB Figure S3: Control groups for the effect of DMSO on memory retention dynamics; Figure S4: Learning and memory comparison between FVB and C57Bl/6J mice; Table S1: The number of peptides (n) identified by mass spectroscopy with 15N/14N ratio > 0.01.
