# Supplementary material for: Do Perineuronal Nets Stabilize the Engram of a Synaptic Circuit?
Source: Cells. 2024 Sep 29;13(19):1627. doi: 10.3390/cells13191627 (PMC11476018; doi:10.3390/cells13191627)
Supplement: Supplementary file 1 [file cells-13-01627-s001.zip › cells-3082566-supplementary.pdf]

Supplementary figures

Figure S-1

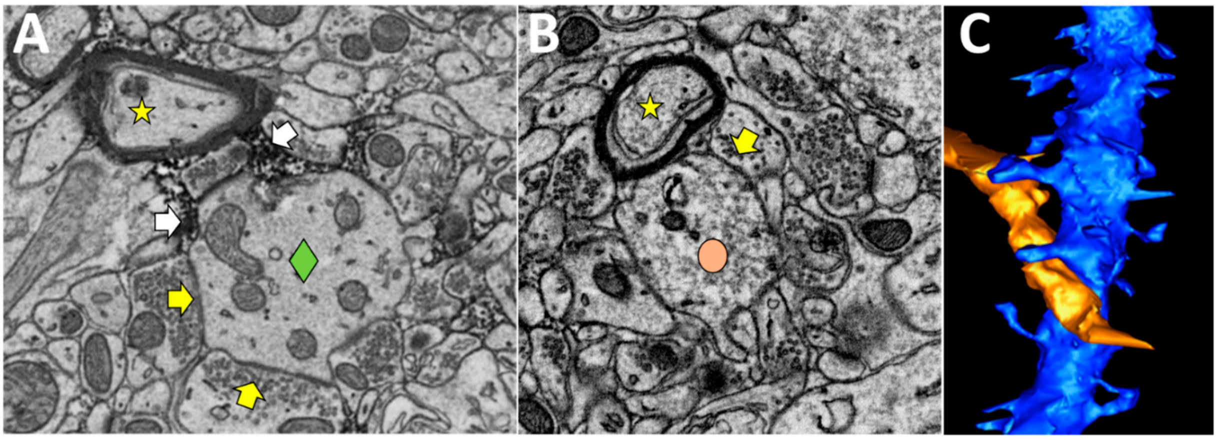

Figure S1. **PNN engulfed dendrite surrounded with PNN and synapses. Myelinated fibers that pass by do not touch the dendrite.**

(**A**) The plasma membrane of a PNN-engulfed dendrite (green diamond) contact, almost exclusively, only pre-synaptic boutons (yellow arrow) or PNN (white arrow).

(**B**) A “naked” spiny dendrite (Orange circle) contacts other cellular elements like a myelinated fiber (yellow asterisk) and passing dendrites.

(**C**) Demonstration of a 3D reconstructed “naked” spiny dendrite (blue) and a passing dendrite (golden) with membrane-to-membrane contact.

Figure S2

A.

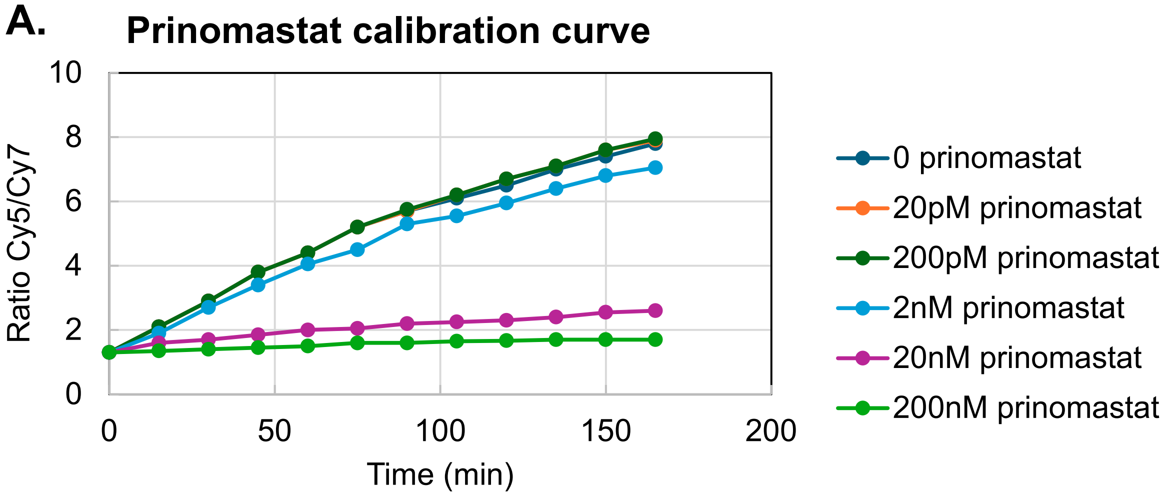

B.

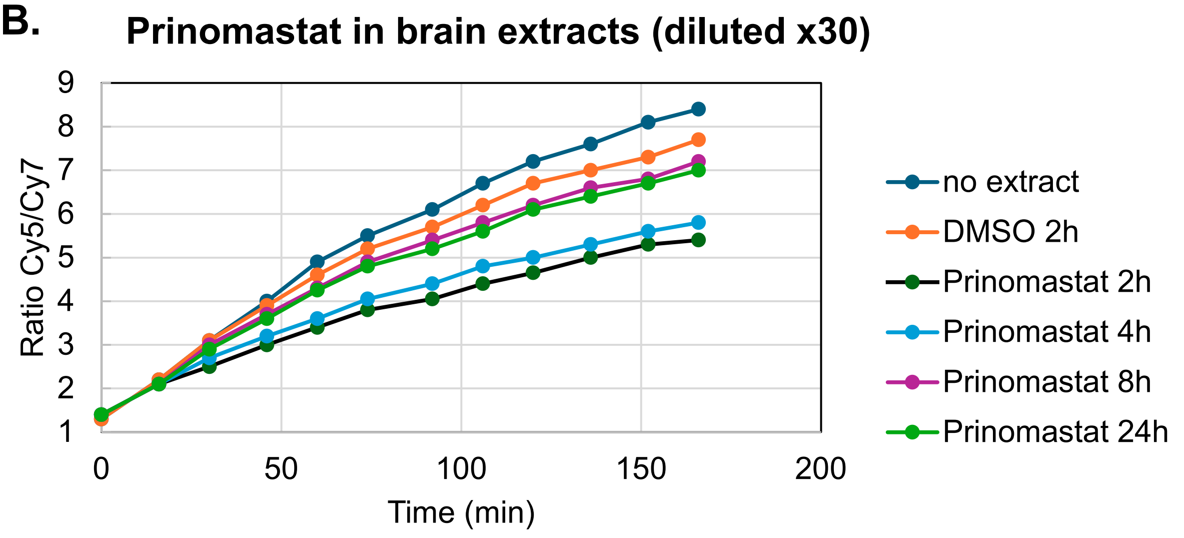

C. Calibration (nM)

Time post injection	Calibration	Brain tissue X30
2 hours	9.6	279
4 hours	8	240
8 hours	2.8	84
24 hours	3.3	99

Figure S2: MMP2 FRET Assay to Determine Whether Prinomastat is Crossing the BBB Figure

Figure S3

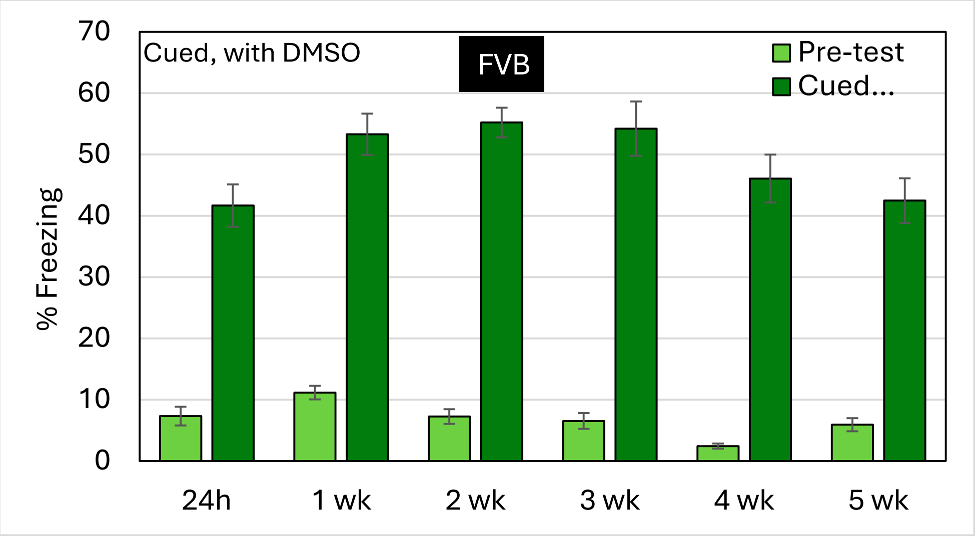

Figure S3. Control groups for the effect of DMSO on memory retention dynamics. In parallel to the prinomastat injection, control cohorts were injected with DMSO at the same time. For each time point, a different group of mice was used to prevent the possible extinction of the fear conditioning. These mice demonstrate the persistence of memory for fear conditioning cue test compared to the prinomastat-injected mice (Figure 4C)

Figure S4

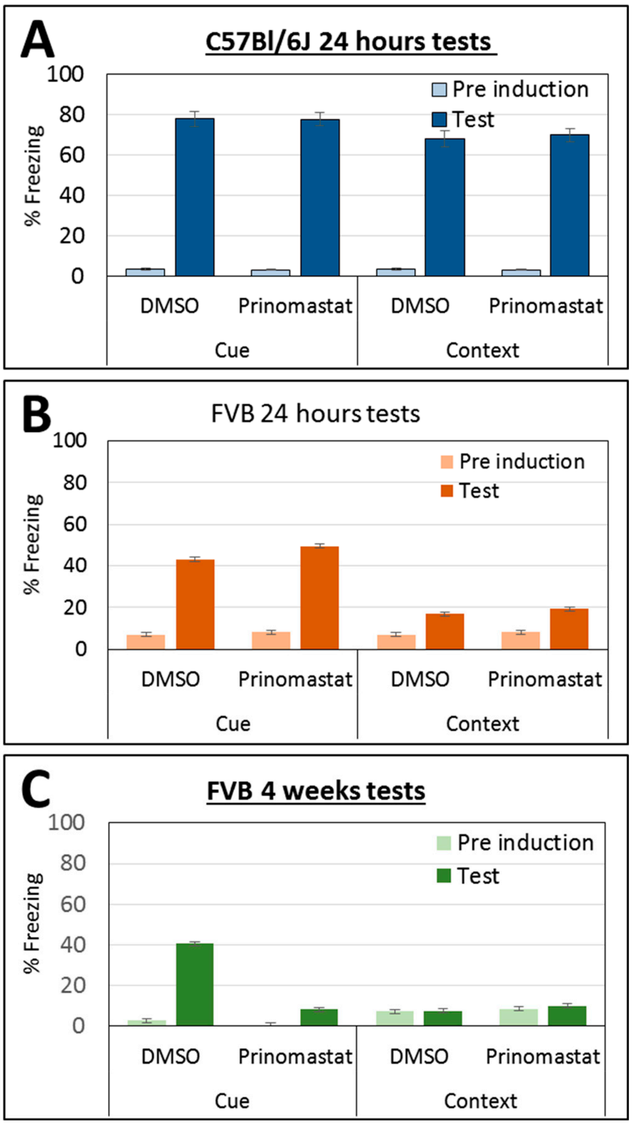

Figure S4. **Learning and memory comparison between FVB and C57Bl/6J mice.** Fear conditioning memory tested 24 hours after induction demonstrates a higher percentage of freezing by C57Bl/6J mice compared to FVB strain (A&B). FVB mice have very little contextual memory, even 24 hours after fear induction (B), and no contextual memory four weeks post-induction, while C57Bl/6J mice acquire (S4A) and retain (Fig. 4D) contextual memory.

Table S1. The number of peptides (n) identified by mass spectroscopy with $^{15}N/^{14}N$ ratio >0.01. These peptides were included in the bar graph of Figure 3

	Conventional cages (6 month pulse -chase)	Enriched environment (6 month pulse - chase)	Conventional cages (18 month pulse - chase)
CamKII α,β,γ,δ	28	40	11
Synapsin 1	38	25	21
Synapsin 2	27	22	5
SynCAM 1,2,3,4	48	28	125
CPEB 2,3,4	6	0	7
Aggrecan	11	25	48
Brevican	57	50	49
Hapln1	32	46	55
Versican	46	32	75
Tenascin-R	99	104	95
Histone 4	40	24	13
Myelin Basic Protein	27	22	94

Table S1. The number of peptides (n) identified by mass spectroscopy with $^{15}N/^{14}N$ ratio >0.01. These peptides were included in the bar graph of Figure 3.
